# Chemistry of Several Sterically Bulky Molecules with P=P, P=C, and C≡P Bond

**DOI:** 10.3390/molecules27051557

**Published:** 2022-02-25

**Authors:** Masaaki Yoshifuji

**Affiliations:** 1 Department of Chemistry, Graduate School of Science, Tohoku University, Sendai 980-8578, Japan; masaaki.yoshifuji.a4@tohoku.ac.jp; masaaki@yoshifuji.org; 2 Department of Chemistry and Biochemistry, College of Arts and Sciences, The University of Alabama, Tuscaloosa, AL 35401, USA; myoshifuji@ua.edu

**Keywords:** steric protection, low-coordinate phosphorus compound, diphosphene, phosphaalkene, phosphaalkyne, phosphaallene, diphosphinidenecyclobutene, catalytic reaction

## Abstract

Several sterically protected, low-coordinate organophosphorus compounds with P=P, P=C, and C≡P bond are described in this study. Molecules such as diphosphenes, phosphaalkenes, 1-phosphaallenes, 1,3-diphosphaallenes, 3,4-diphosphinidenecyclobutenes, and phosphaalkynes are stabilized with an extremely bulky 2,4,6-tri-*t*-butylphenyl (Mes*) group. The synthesis, structures, physical, and chemical properties of these molecules are discussed, together with some successful applications in catalytic organic reactions.

## 1. Introduction

Low-coordinate organophosphorus compounds with coordination numbers of 1 or 2 were once believed to only exist as unstable species. However, by introducing a sterically bulky group such as 2,4,6-tri-*t*-butylphenyl into a molecule, various kinds of unusual phosphorus compounds have been isolated as kinetically stable species since 1981. In this review, the characteristics of several compounds carrying P=P, P=C, and P≡C bonds—including the preparation, structural, physical, theoretical and chemical aspects, as well as some catalytic applications to organic reactions—are described.

## 2. Steric Protection and the First “Phosphobenzene”

### 2.1. Steric Protection for Stabilization of Unstable Compounds

**Scheme 1:**
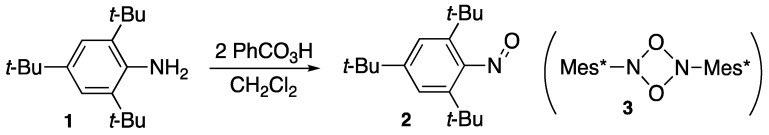
A sterically protected nitrosobenzene (**2**); Mes* = 2,4,6-*t*-Bu_3_C_6_H_2_.

As shown in Scheme 1, Okazaki and Inamoto [1] found that 2,4,6-tri-*t*-butylaniline (**1**) is oxidized with 2 moles of perbenzoic acid in dichloromethane to yield the corresponding nitrosobenzene (**2**), which is stable in air at room temperature and can be purified by column chromatography with alumina. In contrast to normal nitroso compounds such as unsubstituted nitrosobenzene, compound **2** is emerald green and does not dimerize to 1,3,2,4-dioxadiazetidine (**3**) either in solution or the solid state due to the steric hindrance. In general, the steric protection technique seems promising for the stabilization of unstable compounds.

**Scheme 2:**
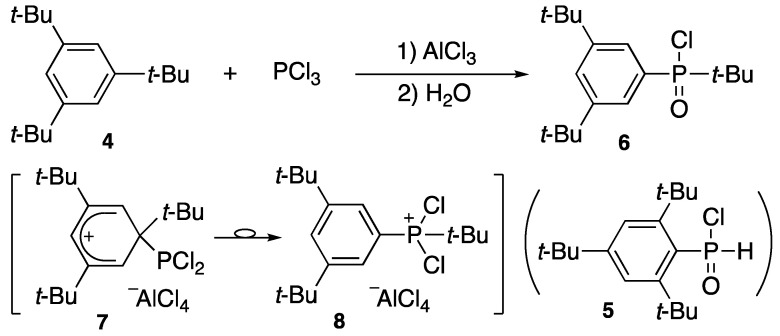
Friedel–Crafts-type reaction of 2,4,6-tri-*t*-butylbenzene (**4**).

In an attempt to introduce a phosphorus moiety to the phenyl ring, phosphorus trichloride was allowed to react with 1,3,5-tri-*t*-butylbezene (**4**) in the presence of aluminum chloride. As reported by Cook [2], in place of the expected 2,4,6-tri-*t*-butylphenylphosphinic chloride (**5**), after partial hydrolysis, *t*-butyl-3,5-di-*t*-butylphenylphosphinic chloride (**6**) was obtained, indicating that one of the *t*-butyl groups migrated from the aromatic ring to the phosphorus atom under Friedel–Crafts reaction conditions, as shown in Scheme 2 [3]. It turned out that *t*-butyl is prone to move around on the ring to rearrange the original positions under the strong acidic conditions, probably due to a labile *t*-butyl cation, e.g., **7** and **8**. Therefore, another strategy was needed to introduce a phosphorus functional group on a bulky benzene nucleus to utilize 2,4,6-tri-*t*-butylphenyl as a sterically demanding group (hereafter abbreviated as Mes*).

### 2.2. Preparation and Characterization of Diphosphenes

**Scheme 3:**
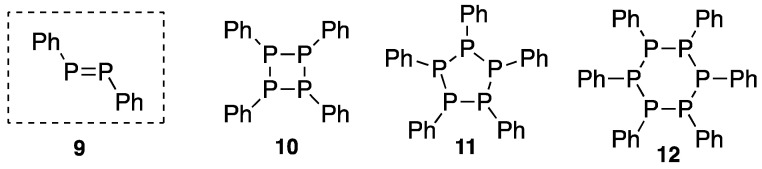
“Phosphobenzene”.

“Phosphobenzene (**9**)” was reported by Köhler and Michaelis [4] as the phosphorus analogue of azobenzene. Later, however, it was revealed that the structure of this compound **9** comprised oligomers such as **10** [5,6], **11** [7,8], and **12** [9,10] according to the molecular weight determination and X-ray analysis (Scheme 3). Since then, it has been believed that such compounds with double bonds involving heavier main group elements never existed as stable compounds.

**Scheme 4:**
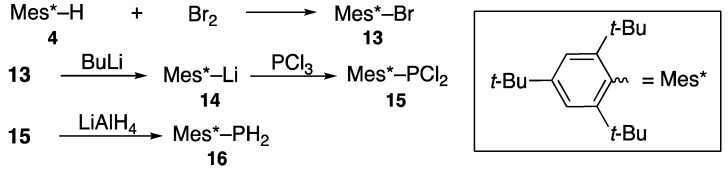
Preparation of sterically bulky phosphonous dichloride **15** and phosphane **16**.

As shown in Scheme 4, 1,3,5-tri-*t*-butylbezene (**4**) is brominated to yield 1-bromo-2,4,6-tri-*t*-butylbenzene (**13**) and is converted to 2,4,6-tri-*t*-butylphenylphosphonous dichloride (**15**) after a halogen–metal exchange with butyllithium, thus yielding **14** followed by the addition of phosphorus trichloride. Dichloride **15** reacts with lithium aluminum hydride to yield 2,4,6-tri-*t*-butylphenylphosphane (**16**). It should be noted that both bulky phosphorus compounds **15** and **16** are stable, easy to handle in air at room temperature, and can serve as appropriate starting materials for the study of low-coordinate organophosphorus compounds [11].

**Scheme 5:**
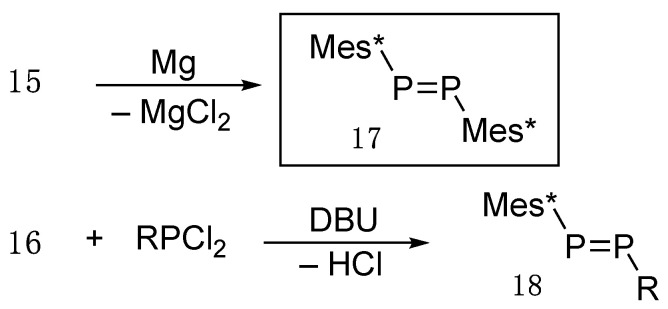
Preparation of diphosphenes (**17** and **18**); DBU = 1,8-diazabicyclo[5.4.0]undec-7-ene.

As shown in Scheme 5, dichloride **15** is allowed to react with magnesium in THF to yield a stable diphosphene **17** as an orange red crystalline material with an mp of 175–176 °C [12]. Some selected physical and X-ray data are listed in Table 1. It is noteworthy that (*E*)-bis(2,4,6-tri-*t*-butylphenyl)diphosphene (**17**), which turned out to be a true “phosphobeneze,” has a short P–P bond distance (2.034(2) Å) and shows a low ^31^P NMR chemical shift (*δ*_P_ 492.4 ppm).

According to the crystallographic analysis, the bond distance between the two phosphorus atoms is about 10% shorter than the regular P–P single bond, and the dihedral angle ∠C–P–P–C reveals a planar molecular system with (*E*)-configuration.

However, in our initial paper [12], we misreported the ^31^P NMR chemical shift of **17** as *δ*_P_ −59.00 ppm and immediately corrected the value for **17** to 492.4 ppm (C_6_D_6_) because it is one of the most important physical characteristics [13]. In the meantime, Lappert [15] critcized our initially reported chemical shift but Cowley [16] argued our structure of diphosphene itself as diphosphane Mes*P(H)–P(H)Mes* (^31^P NMR *δ*_P_ of −64.4 ppm for *dl* and *δ*_P_ of −65.0 ppm for *meso*) [17], despite our unambiguously determined X-ray analysis [12]. On the other hand, it turned out that the coupling constant between two phosphorus nuclei, ^1^*J*_PP_, is large and has been confirmed by NMR measurement for unsymmetrical diphosphene **18** prepared from the dehydrochlorination reaction of the primary phosphane **16** and RPCl_2_ with a base such as DBU (1,8-diazabicyclo[5.4.0]undec-7-ene) [18] (Scheme 5, second row). Some selected coupling constants and chemical shifts are listed in Table 2 [19].

**Scheme 6:**
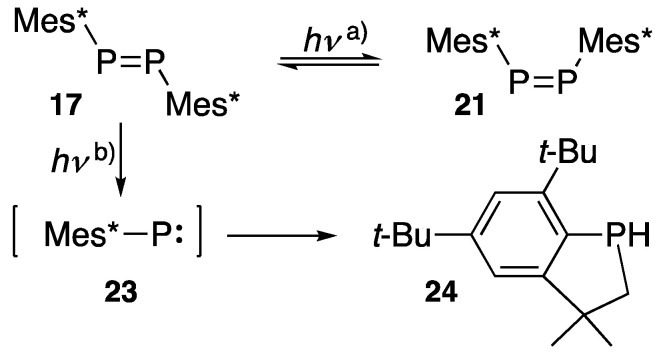
Temperature- and wavelength-dependent photolysis of diphosphene **17**: (**a**) Hg lamp irradiation through a Pyrex filter or Ar-laser irradiation at low temperature; (**b**) Hg lamp without a Pyrex filter.

^31^P NMR data for (*Z*)-diphosphenes **21** (*δ*_P_ 368 ppm) and **22** (*δ*_P_ 394 ppm) show a higher chemical shift than (*E*)-diphosphenes. As shown in Scheme 6, the formation of (*Z*)-bis(2,4,6-tri-*t*-butylphenyl)diphosphene (**21**) is observed during the temperature- and wavelength-depending photolysis of (*E*)-diphosphene **17**. The irradiation of **17** with a Hg lamp through a Pyrex filter at –40 °C [26], or argon-laser irradiation (514.5 nm) at −78 °C [27] yields an *E/Z* equilibrium mixture that returns to *E*-form upon warming, showing that (*Z*)-diphosphene **21** is thermally unstable due to the steric congestion caused by the two adjacent Mes* groups. Caminade reported the first order rate constant of *E/Z* isomerization reaction as Δ*G*^≠^_273_ = 20.35 kcal/mol based on ^31^P-NMR studies. When the photolysis is conducted without a Pyrex filter, regardless of the temperature between −78 °C and 0 °C [26], intramolecular insertion to a neighboring methyl group occurs and yields benzophosphaindane **24**, most likely suggesting the generation of phosphinidene intermediate **23** during the photolysis.

In the course of the synthetic route from **15** to **17** (Scheme 5, first row), a “free” **23** does not seem to be generated since Protasiewicz failed to obtain **17** under controlled reaction conditions such as when employing extremely dried THF with activated magnesium metal [29], instead yielding **24**.

**Scheme 7:**
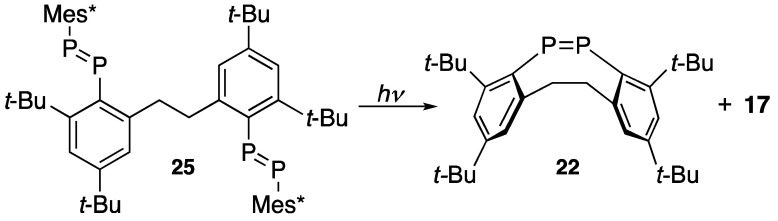
Ring closing metathesis of bis(diphosphene) **25** by Xe-lamp irradiation.

Scheme 7 shows that (*Z*)-diphosphene **22** is prepared via photolytic ring closing metathesis (RCM) on bis(*E*-diphosphene) **25** [28] with a Xe-lamp in benzene for 30 min at room temperature, though the yield of **22** is not satisfactory.

It should be noted that in the year of 1981, when a stable diphosphene **17** was isolated for the first time, West reported the successful isolation of a stable disilene (Mes_2_Si = SiMes_2_; Mes = 2,4,6-Me_3_C_6_H_2_) protected with four mesityl groups [30]. The molecular structures of these two compounds are simple but unusual and uncommon based on the traditional knowledge of organic, inorganic, and/or physical chemistry [31,32,33,34,35,36,37]. Since then, however, various kinds of sterically protected stable low-coordinate phosphorus compounds have been successfully prepared and characterized. Many sophisticated or simple protective groups other than Mes* with stronger or weaker steric ability, with or without electronic effect, or for special or common purposes have been developed, and some early examples have already been reviewed [18,38,39].

### 2.3. Chemical Reactivity of Diphosphenes

Diphosphenes demonstrate a variety of chemical reactions including photolysis, oxidation, reduction, hydrolysis, sulfurization, carbene-addition, and coordination to transition-metals [31,32]. In this section, selected examples are described in more detail.

**Scheme 8:**
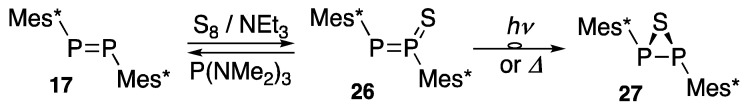
Reaction of diphosphene **17** with sulfur.

Diphosphene **17** is allowed to react with sulfur in triethylamine at room temperature overnight to yield a stable diphosphene mono-sulfide **26**, and the desulfurization to the starting diphosphene **17** is carried out with hexamethylphosphorous triamide in benzene at room temperature for 2 h. Sulfide **26** is converted to thiadiphosphirane **27** upon the irradiation of light with a medium-pressure Hg lamp at 0 °C for 5 min or heating in toluene for 30 min at 95 °C (Scheme 8) [40,41].

**Scheme 9:**
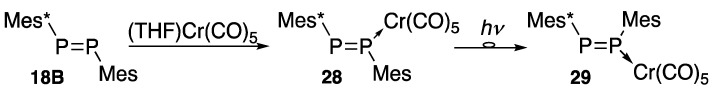
Reaction of diphosphene **18B** with carbonylchromium(0); Mes = 2,4,6-Me_3_C_6_H_2_.

Unsymmetrical diphosphene **18B** is allowed to react with (THF)Cr(CO)_5_ to yield an end-on complex **28** as a result of coordination at the less hindered site. However, complex **28** is converted to isomer **29** upon the irradiation of light with a medium-pressure Hg lamp in hexane at 0 °C for 5 min due to the *E/Z* isomerization around the P=P bond. The *Z*-ligand on chromium in **29** is thermally stable, probably because the steric environment reduces the enthalpy change between **28** and **29**, in contrast to that of free ligand **18B** (Scheme 9) [42].

## 3. Stable Phosphaalkenes and Phosphaalkynes

### 3.1. Stable Phosphaalkenes and the Related Compounds

**Scheme 10:**
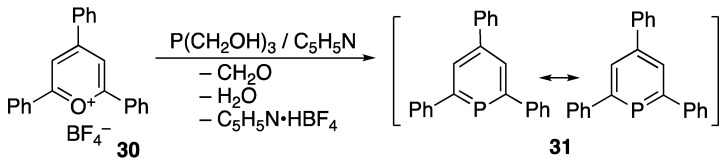
Preparation of phosphabenzene **31**.

In 1966, Märkl reported the interesting preparation of a stable phosphorus-containing heterocyclic compound from 2,4,6-triphenylpyrylium tetrafluoroborate (**30**) [43]. The obtained triphenylphosphabenzene (or phosphinine) (**31**) contains a P=C bond in its canonical form, but the stability is estimated to be due to delocalization or aromaticity (Scheme 10).

**Scheme 11:**
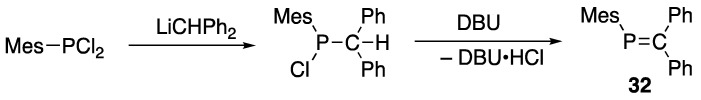
Preparation of phosphaethene **32**; Mes = 2,4,6-Me_3_C_6_H_2_; DBU = 1,8-diazabicyclo[5.4.0]undec-7-ene.

Later in 1978, Bickelhaupt reported a thermally stable phosphaalkene **32** with a localized P=C double bond that is sterically protected with mesityl group [44], as shown in Scheme 11. This successful isolation of **32** triggered our interest in the field of sterically protected, low-coordinate phosphorus compounds.

**Scheme 12:**
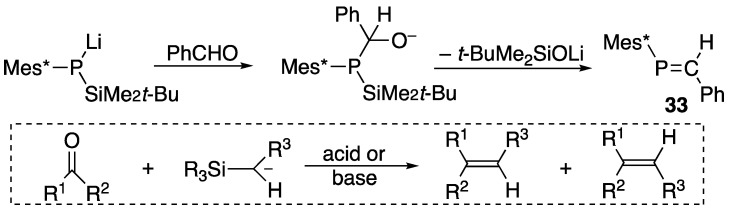
Preparation of phosphaalkene **33** and the Peterson reaction.

Alternatively, more sterically protected phosphaethenes with Mes* around phosphorus can be prepared by another method such as the phospha-Peterson reaction starting from silylphosphide and carbonyl compounds, thus successively preparing (*E*)-2-phenyl-1-(2,4,6-tri-*t*-butylphenyl)-1-phosphaethene (**33**), as shown in Scheme 12 [45].

**Scheme 13:**
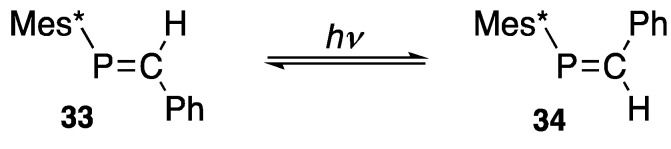
Photoisomerization of a phosphaethene.

Interestingly, phosphaethene **33** in the (*E*)-form is isomerized in benzene upon the irradiation of light with a 100 W medium pressure Hg lamp at 0 °C for 6 h to yield an equilibrium mixture (3:7) with the corresponding (*Z*)-phosphaethene **34** [45], as shown in Scheme 13. After separation with silica-gel column chromatography, both isomers were analyzed by X-ray crystallography [46], and it was found that separated (*Z*)-phosphaethene **34** is thermally stable and does not isomerize to **33** even it is heated at 100 °C in toluene for 24 h.

Table 3 shows ^31^P-NMR chemical shifts for some selected phosphaalkenes. Aromatic phosphorus in **31** shows a higher shift than isolated phosphaalkenes, and (Z)-isomer **34** shows a slightly higher chemical shift than (*E*)-isomer **33**.

**Scheme 14:**
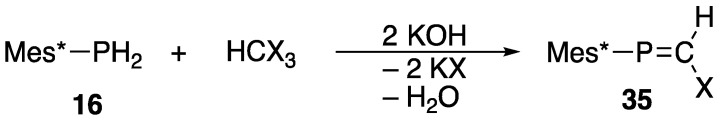
Preparation of 2-halophosphaethene **35**.

Alternatively, Appel reported a convenient method for the preparation of useful 2-halo-1-(2,4,6-tri-*t*-butylphenyl)phosphaethene (**35**) starting from 2,4,6-tri-*t*-butylphenylphosphane (**16**) and trihalomethanes, as shown in Scheme 14 [57].

### 3.2. Some Reactions of Phosphaalkenes

**Scheme 15:**
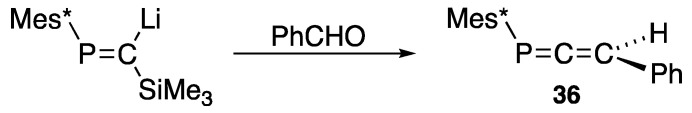
Preparation of phosphaallene **36** via the Peterson reaction.

The Peterson method can be applied to prepare 3-phenyl-1-(2,4,6-tri-*t*-butylphenyl)-1-phosphaallene (**36**), as shown in Scheme 15 [47], indicating that the extended P=C system (phosphacumulenes) is also stable once sterically protected.

**Scheme 16:**
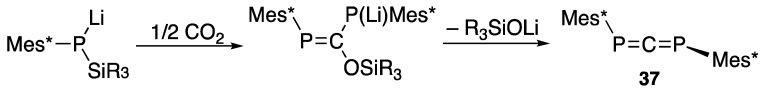
Preparation of diphosphaallene **37** with the phospha-Peterson reaction.

On the other hand, when a silylphosphide is allowed to react with half of an equivalent of carbon dioxide, 1,3-bis(2,4,6-tri-*t*-butylphenyl)-1,3-diphosphaallene (**37**) is formed [48,58] via the phospha-Peterson reaction, as shown in Scheme 16 [47].

**Scheme 17:**
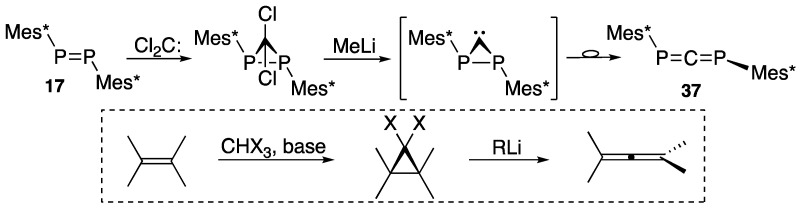
Preparation of diphosphaallene **37** with dichlorocarbene and the Doering–Moore–Skattebøl reaction.

It is interesting to note that diphosphaallene **37** can be alternatively prepared from diphosphene **17** and dichlorocarbene followed by methyllithium with the Doering–Moore–Skattebøl-type reaction (Scheme 17) [20,59], probably via a bracketed carbenoid intermediate. Carbene can be generated in aqueous medium with the Makosza method [60], in the presence of benzyltriethylammonium chloride in a mixture of aqueous 50% NaOH, trichloromethane, and hexane.

**Scheme 18:**
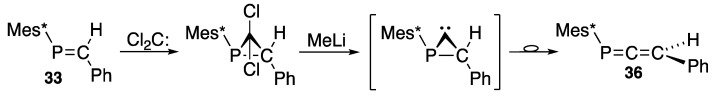
Preparation of phosphaallene **36** with dichlorocarbene.

This one-carbon homologation method [61] can also be applied to prepare phosphaallene (**36**), as shown in Scheme 18 with a combination of **33** and dichlorocarbene [59].

Phosphacumulenes **36** and **37** have axial dissymmetry according to their X-ray structures [62,63,64], so, under achiral circumstances, the reaction products are expected to be a 1:1 mixture of enantiomers, though in Scheme 15, Scheme 16, Scheme 17 and Scheme 18 for **36** and **37**, only the (*S*)-isomers were displayed. Actually both are mixtures of enantiomers and could be separated with high-performance liquid chromatography (HPLC). Each separated enantiomer is racemized on exposure to light due to the rotation around the axis of the molecules [47,65], but they were found to be thermally stable in the dark at room temperature, even at 50 °C for 15 h for **36**.

**Scheme 19:**
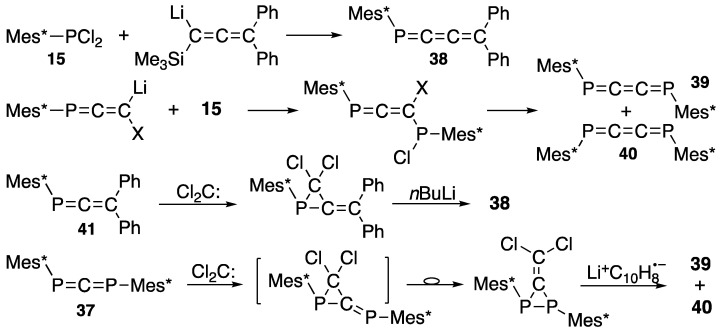
Preparation of phospha-1,2,3-butatrienes (**38**–**40**).

As shown in Scheme 19 (third and fourth rows), the Doering–Moore–Skattebøl method can be applied to the P=C=C and P=C=P systems (**41** and **37**) to yield 1-phospha-1,2,3-butatriene **38** [49] and 1,4-diphospha-1,2,3-butatrienes (**39** and **40**, *ca* 4:1) [51], respectively, which implies that two carbon atoms are inserted into P=C and P=P in a stepwise manner. Additionally, Märkl described the preparation of extended cumulenes (**38**–**40**) from 2,4,6-tri-*t*-butylphenylphosphonous dichloride (**15**) and allenyllithium compounds as nucleophiles (Scheme 19, first and second rows) [50,52].

**Scheme 20:**
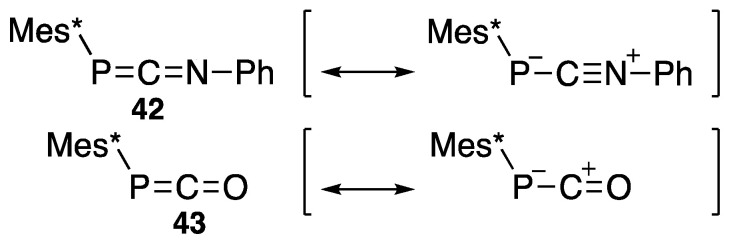
Canonical forms for oxygen- and nitrogen-incorporated phosphacumulenes.

Table 3 also lists P=C-containing compounds such as **42** [53] and **43 [54]**, whose chemical shifts have higher values, which could be rationalized if their canonical forms are considered as depicted in Scheme 20, where the anion is located on the phosphorus atom.

### 3.3. Stable Phosphaalkynes

**Scheme 21:**
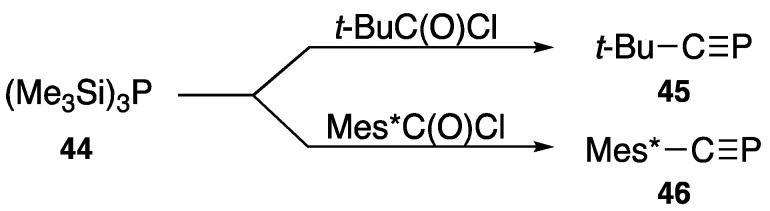
Preparation of phosphaalkynes (**45** and **46**).

Phosphaalkynes have the lowest coordination number of 1, and once sterically protected, this type of compound can be isolated. Using tris(trimethylsilyl)phosphane (**44**) and acid chlorides, Becker [55] reported the *t*-butyl derivative **45** in 1981, as shown in Scheme 21. With a similar method, Märkl [56] described the 2,4,6-tri-*t*-butylphenyl derivative **46** in 1986.

**Scheme 22:**
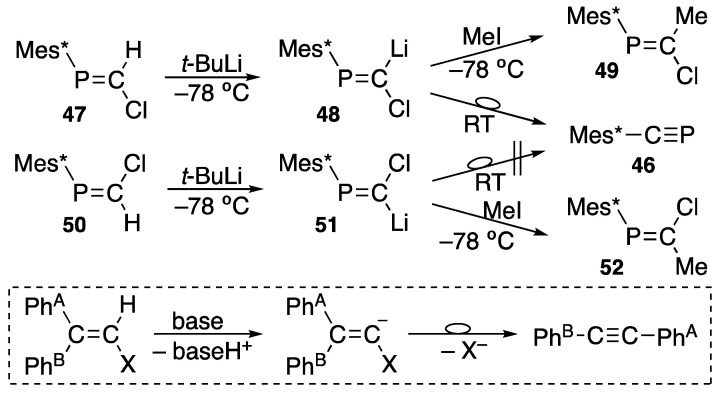
Preparation of phosphaalkyne **46** and the Fritsch–Buttenberg–Wiechell reaction; RT = room temperature.

Alternatively, phosphaethyne (**46**) can be prepared by an interesting rearrangement reaction, a phosphorus version of the Fritsch–Buttenberg–Wiechell rearrangement, as shown in Scheme 22 [66,67,68]. (*E*)-2-Chloro-1-(2,4,6-tri-*t*-butylphenyl)phosphaethene (**47**) is metalated to **48** with *t*-butyllithium at −78 °C, and the metalation is confirmed by methylation with iodomethane at that temperature to yield (*E*)-2-chloro-1-(2,4,6-tri-*t*-butylphenyl)-1-phosphapropene (**49**). However, after metalation, when **48** is allowed to warm up to room temperature, phosphaethyne **46** is obtained, indicating that the Mes* group migrates from phosphorus to carbon. On the other hand, if (*Z*)-2-chloro-1-(2,4,6-tri-*t*-butylphenyl)phosphaethene (**50**) is allowed to react with *t*-butyllithium, the obtained lithium compound (**51**) does not lead to phosphaethyne **46**, while the formed alkylation product, (*Z*)-2-chloro-1-(2,4,6-tri-*t*-butylphenyl)-1-phosphapropene (**52**), indicates that the configuration is retained [67]. Based on a kinetic study of the Fritsch–Buttenberg–Wiechell rearrangement shown at the bottom of Scheme 22, Köbrich [69] suggested that phenyl group Ph^A^ migrates from one carbon to another, thus eliminating *trans*-halogen atom as halide to form acetylene [70].

**Scheme 23:**
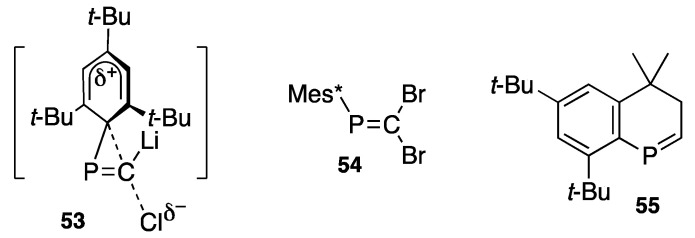
A plausible transition state (**53**) and compounds **54** and **55**.

**Scheme 24:**
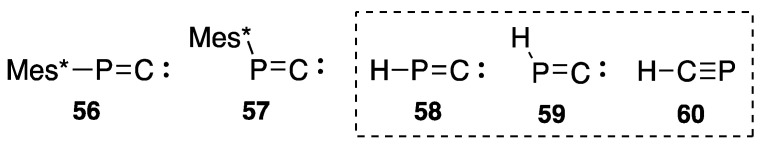
Structures of phosphanylidene carbenes and phosphaalkynes.

In this rearrangement process from **47** to **46**, a similar transition state such as **53** might be involved (Scheme 23). Appel [71] reported the formation of **46** from 2,2-dibromo-1-(2,4,6-tri-*t*-butylphenyl)phosphaethene (**54**), though it is not certain if phosphanylidenecarbenes such as **56** or **57** (Scheme 24) are involved in this formation of phosphaalkyne, whereas the carbene, if freely generated, is experimentally [72] and theoretically [73] known to yield an intramolecular insertion product such as **55**. 

Concerning the energy difference between phosphanylidenecarbene (linear **56** or bent **57**) and phosphaethyne **46**, we calculated total energy with an ab initio method and found that **46** has total energy of 87.3 kcal/mol less than that of **56** or **57** [70]. Additionally, in 1986, Nguyen theoretically computed 83.9 kcal/mol as the difference between **58** and **60** in a model system [74], and Lehmann [75] calculated the difference between **59** and **60** as 84.1 kcal/mol in 1985. Each computed result indicates that phosphaalkynes are far more stable than the corresponding phosphanylidenecarbene isomers (Scheme 24).

### 3.4. Singlet Biradicals from Phosphaalkyne

**Scheme 25:**
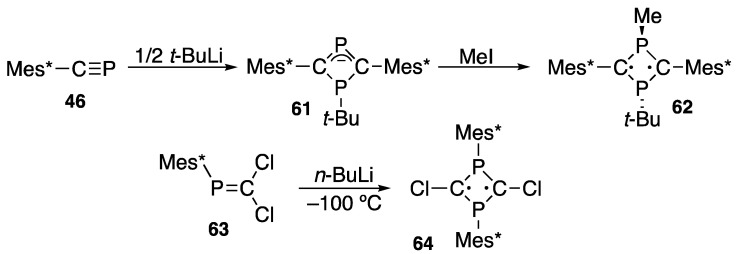
Two types of stable singlet biradicals (**62** and **64**).

An interesting reactivity of phosphaalkyne **46** (with the lowest coordination number of 1) is dimerization promoted by half of an equivalent of *t*-butyllithium to a singlet biradical species such as 1-*t*-butyl-3-methyl-2,4-bis(2,4,6-tri-*t*-butylphenyl)-1,3-diphosphacyclobutane-2,4-diyl (**62**) after quenching **61** with iodomethane (Scheme 25) [76,77]. In 1995, Niecke reported 2,4-dichloro-1,3-bis(2,4,6-tri-*t*-butylphenyl)-1,3-diphosphacyclobutane-2,4-diyl (**64**) [78] as a reductive coupling reaction product of 2,2-dichloro-1-(2,4,6-tri-*t*-butylphenyl)phosphaethene (**63**) at −100 °C. The radicals are on the carbon atoms in both cases, but the Mes* groups are contrarily located. An attempt to combine these two different singlet biradicals was described for a novel radical system [79].

Cyclic phosphide anion **61** serves as a good nucleophile to yield various kinds of biradical species in addition to methyl derivative **62**. Anion **61** reacts with several arynes or benzynes to yield phenyl-substituted biradicals [80]. In place of the *t*-butyllithium shown in Scheme 25, a series of carbon nucleophiles (including *t*-butyl iodide and even nitrogen nucleophiles such as LDA (lithium diisopropylamide)) can be used [77]. It should be noted that nucleophiles initially attack the phosphorus atom to form **61**, in contrast to the corresponding nitriles.

**Scheme 26:**
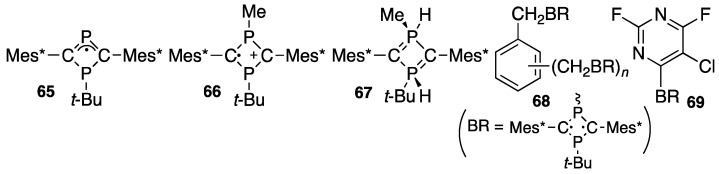
Some singlet biradical-related species.

Compound **62** is a deep blue solid with an mp of 158–160 °C and thermally stable even in a solution in air. The ^31^P NMR spectrum shows an ABq at *δ*_P_ 55.9 (*t*-BuP) and −11.3 (MeP) ppm with ^2^*J*_PP_ 362.8 Hz, and the ^13^C NMR spectrum of the ring C shows a dd at *δ*_C_ 111.3 (ring C) with ^1^*J*_CP_ 10.7 and 3.3 Hz. An X-ray study indicated no apparently direct bonding between either P–P (2.43 Å) or C–C (2.50 Å) in the ring with two almost perfectly planar carbon atoms. No signal was observed in EPR measurements, indicating that the compound is a singlet biradical species. The chemical reactivity of singlet biradicals is of high interest, including hydrolysis with water [77], oxidation with molecular oxygen [77], TEMPO (2,2,6,6-tetramethyl-1-piperidinoxy) [77], and ammoniumyl antimonate [81] yielding a radical cation (**66**), sulfurization with elemental sulfur [82], reduction with hydride (**67**) [83,84], muonium addition [85], respectively. Oligomeric poly-biradical species **68** [86] can be prepared and pyrimidine derivatives such as **69** are promising for HF-capture reagents [87]. The formation of neutral radical **65**, which is obtained via the partial oxidation of cyclic phosphide anion **61** with iodine, is also noteworthy. The radical is stable in air, and X-ray analyses, theoretical calculations, and EPR spectroscopic studies have indicated that **65** is a carbon-centered radical [88] (Scheme 26).

## 4. Diphosphinidenecyclobutenes

### 4.1. Preparation of Diphosphinidenecyclobutenes

**Scheme 27:**
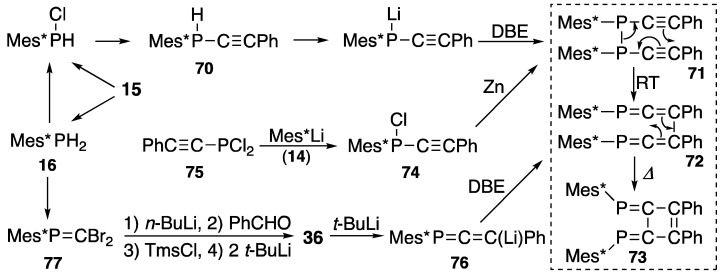
Preparation of diphosphinidenecyclobutene **73**; DBE = 1,2-dibromoethane, RT = room temperature.

**Scheme 28:**
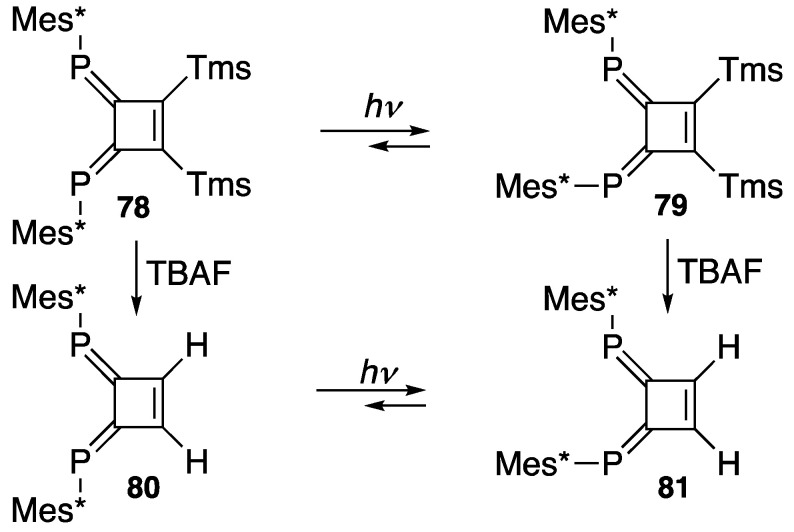
*E/Z* photoisomerization of **78** and **80**; TBAF = *n*-Bu_4_N^+^F^−^.

Diphosphinidenecyclobutenes, abbreviated as DPCBs, are prepared as depicted in Scheme 27. From 2,4,6-tri-*t*-butylphenylphosphane (**16**) or 2,4,6-tri-*t*-butylphenylphosphonous dichloride (**15**), (2-phenylethynyl)(2,4,6-tri-*t*-butylphenyl)phosphane (**70**) is prepared via chloro(2,4,6-tri-*t*-butylphenyl)phosphane. After metalation followed by oxidative coupling with 1,2-dibromoethane (DBE) at low temperature, the corresponding diphosphane **71** is formed, thus leading to 1,6-diphospha-1,2,4,5-hexatetraene **72** at room temperature through a Cope rearrangement. Upon heating, tetraene **72** rearranges to (*E*,*E*)-1,2-diphenyl-3,4-bis(2,4,6-tri-*t*-butylphenylphosphinidene)cyclobutene (**73**) [89,90]. Compound **73** was described by Appel in 1987 [91], and the corresponding 3,4-bis(trimethylsilyl) derivative (**78** in Scheme 28) was prepared by ourselves with this phospha-Cope method [92]. Phosphinidenecyclobutene **73** can be prepared in other routes, e.g., as shown in the middle row of Scheme 27, from (2-phenylethynyl)(2,4,6-tri-*t*-butylphenyl)phosphinous chloride (**74**) followed by reductive coupling reaction that forms **71**, whereas **74** is prepared from 2-phenylethynylphosphonous dichloride (**75**) and 2,4,6-tri-*t*-butylphenyllithium (**14**); as shown at the bottom of Scheme 27, from (1-phosphaallen-3-yl)lithium **76** followed by oxidative coupling reaction with DBE forms **72** [89,90]; and Scheme 15 suggests that, 1-phosphaallene **36**, as a precursor of **76**, can be prepared with the Peterson reaction of 2,2-dibromo-1-(2,4,6-tri-*t*-butylphenyl)phosphaethene (**77**) with benzaldehyde.

As shown in Scheme 13 for phosphaethene **33**, (*E*,*E*)-3,4-bis(2,4,6-tri-*t*-butylphenylphosphinidene)-1,2-bis(trimethylsilyl)cyclobutene (**78**) can undergo *E/Z* photoisomerization and yield (*E,Z*)-isomer **79** (Scheme 28). A simple DPCB **80**, which is derived from **78** and tetrabutylammonium fluoride (TBAF), also shows a similar photoisomerization for **81**. It should be noted that in both cases, even after a longer irradiation time, the (*Z,Z*)-isomer is not formed, probably due to the severe congestion around the adjacent Mes* groups [93].

**Scheme 29:**
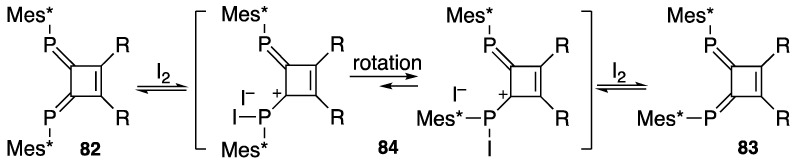
Isomerization from **82** to **83** induced by iodine; R = Ph, Tms, H.

It is noteworthy that iodine initiates *E/Z* isomerization, as shown in Scheme 29. (*E,E*)-DPCB **82** was found to be isomerized to the corresponding (*E,Z*)-isomer **83** in the presence of iodine in THF at room temperature for 1 h [94]. Facile rotation between phosphorus and carbon in cyclobutenylium iodide **84** could operate as an intermediate during the process of isomerization.

**Scheme 30:**
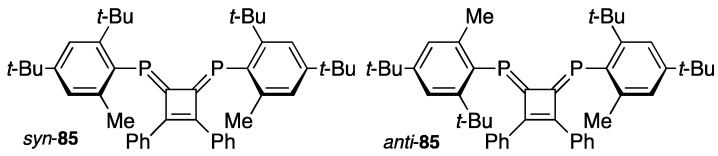
Rotational isomers *syn-* and *anti*-85.

Bulky groups such as Mes* on phosphorus atoms appear to suffer from free rotation around the P–C bond and thus hinder the rotation that distinguishes *syn-* and *anti*-rotamers for DPCB **85**, which is protected with two unsymmetrical substituents such as 2,4-di-*t*-butyl-6-methylphenyl (Scheme 30). Two types of the tetracarbonyltungsten complexes of **85** were separated by column chromatography, and the *syn*-isomer could be analyzed by X-ray crystallography. The *anti*-isomer was further analyzed with a chiral HPLC column, and it was revealed that the isomer consists of two enantiomers, which was confirmed with the CD spectra [95].

**Scheme 31:**
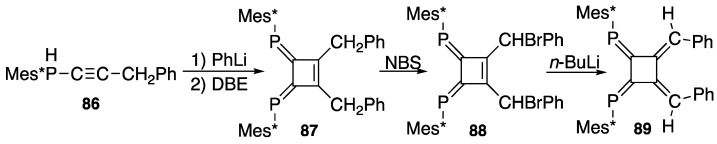
Preparation of diphospha[4}radialene **89**; DBE = 1,2-dibromoethane, NBS = *N*-bromosuccinimide.

As an interesting derivative related to DPCB, 1,2-diphospha[4]radialene **89** is prepared as shown in Scheme 31 from 1,2-dibenzyl-DPCB **87**. (3-Phenylprop-1-yn-1-yl)(2,4,6-tri-*t*-butylphenyl)phosphane (**86**) is lithiated with phenyllithium, followed by oxidative coupling with 1,2-dibromoethane (DBE) to yield 1,2-dibenzyl DPCB **87**, which is brominated with *N*-bromosuccinimide (NBS) to yield bis(α-bromobenzyl) derivative **88**; finally, a reaction with butyllithium at −78 °C yields (*E,E,E,E*)-1,2-dibenzylidene-3,4-bis[(2,4,6-tri-*t*-butylphenyl)phosphinidene]cyclobutane (**89**). The configuration was confirmed with X-ray analysis; **89** appears to suffer from steric crowding between the two phenyl groups by taking the *E*-configuration, though they tilt about by 30° from the radialene plane in the same direction, while the Mes* groups are almost perpendicular to the plane so they avoid congestion within the molecule at least in the solid state [96].

### 4.2. Transition Metal Complexes of Diphosphinidenecyclobutenes

**Scheme 32:**
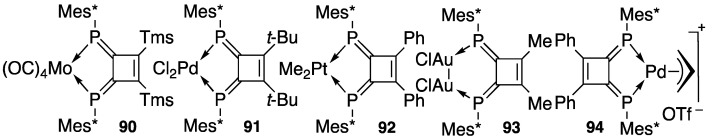
Some DPCB–metal complexes; Tf = CF_3_SO_2_.

As expected, DPCB **82** is a good bidentate ligand for transition metals such as chromium, molybdenum, tungsten, iron, gold, palladium, platinum, rhodium. Scheme 32 shows several typical examples (**90**–**94**) that have been analyzed in X-ray studies [97,98,99,100,101,102,103]. It is noteworthy that the molecular structure of **94** shows that the π-allyl system is perpendicular to the DPCB plane, which controls the stereochemistry in the catalytic reactions. The details of this phenomenon are described later in this section.

**Scheme 33:**
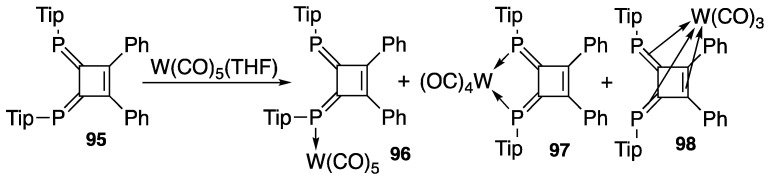
Tungsten carbonyl complexes of **95**; Tip = 2,4,6-*i-*Pr_3_C_6_H_2_.

The reaction of 1,2-diphenyl-3,4-bis(2,4,6-triisopropylphenylphosphinidene)cyclobutene (**95**) with pentacarbonyltungsten leads to an interesting result, as depicted in Scheme 33: it not only serves as a bidentate ligand to yield **97** but also it yields **96** as a monodentate ligand. More interestingly, the ligand serves as a side-on ligand to yield **98** [104].

### 4.3. Synthetic Application to Catalytic Reactions of DPCB–Transition Metal Complexes

**Scheme 34:**
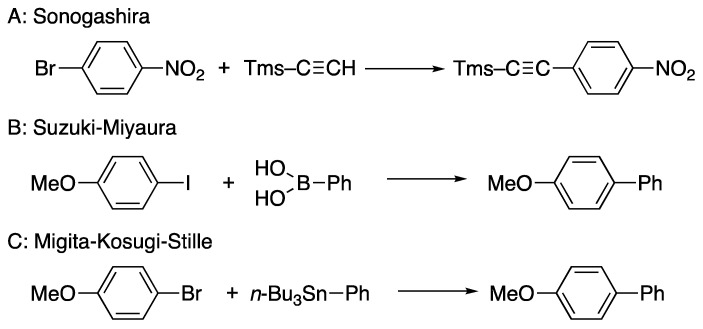
Some cross-coupling reactions catalyzed by DPCB–metal complexes. (**A**) Sonogashira reaction, (**B**) Suzuki–Miyaura reaction, (**C**) Migita–Kosugi–Stille reaction.

DPCB complexes are effective catalysts for organic reactions [36,103,105,106,107]. Scheme 34 demonstrates some of the cross-coupling reactions—such as the Sonogashira [99] (A), Suzuki–Miyaura (B) [105], and Migita–Kosugi–Stille (C) [108] reactions catalyzed by DPCB–transition metal complexes—with representative examples.

**Scheme 35:**
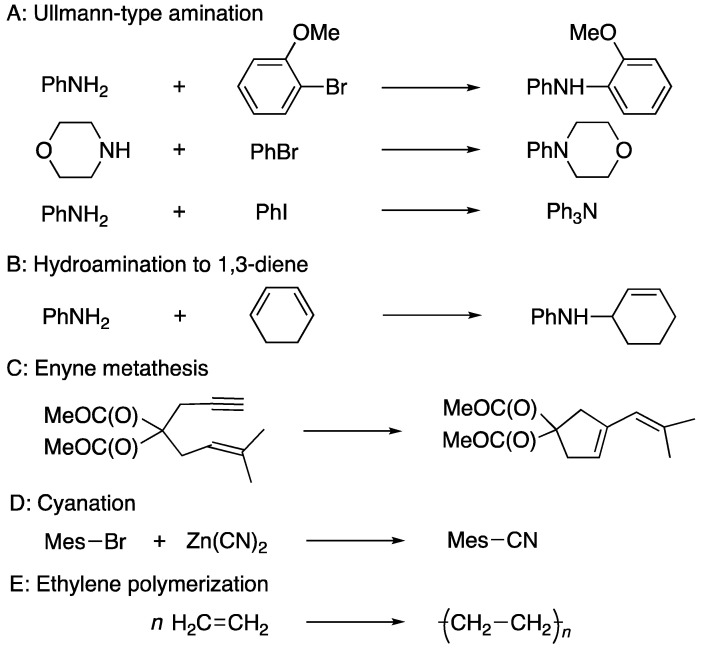
Ullmann-type amination (**A**), hydroamination to 1,3-diene (**B**), enyne metathesis (**C**), cyanation (**D**), and ethylene polymerization (**E**) catalyzed by DPCB–metal complexes. Mes = 2,4,6-Me_3_C_6_H_2_.

Scheme 35 shows some other examples. The Ullmann-type amination reactions (A) can be executed with a DPCB catalyst without a solvent at room temperature [109,110]. Hydroamination to 1,3-dienes (B) can be induced at room temperature without a solvent [103]. An enyne metathesis reaction (C) proceeds via a DPCB–gold complex **93** [101]. Cyanation reactions (D) are widely applied to convert halobenzenes to cyanobenzenes catalyzed by **94** [111]. Ethylene polymerization (E) is catalyzed by thermally stable palladium and a platinum DPCB-ligated catalyst such as **91** and **92** [97,100].

A stable (π-allyl)palladium complex such as **94** (shown in Scheme 32) [103] has shown a wide range of catalytic reactivity including in the Tsuji–Trost-type reaction that enables allylic alcohols to yield amines via the elimination of water [36,105,106] under mild conditions such as room temperature for 1 h with a 0.1 mol% DPCB–palladium catalyst.

**Scheme 36:**
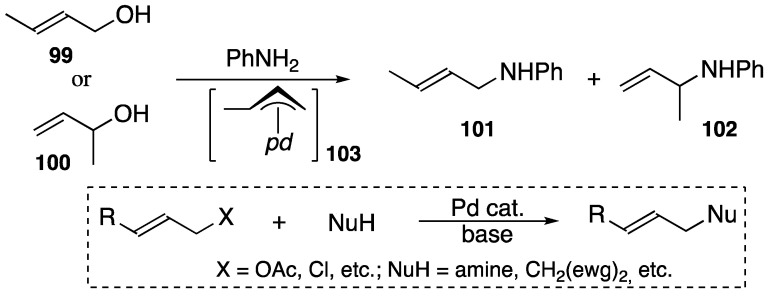
DPCB–palladium-catalyzed amination from C4-allylic alcohols and the Tsuji–Trost reaction; ewg = electron-withdrawing group, *pd* = (DPCB)Pd^+^.

Scheme 36 [112] shows that, starting from either from primary or secondary C4-allylic alcohol (**99** or **100**), products **101** and **102** are regioselectively obtained in the same ratio (86:14) and the same yield (95%), indicating that (π-allyl)palladium complex **103** is a common intermediate.

**Scheme 37:**
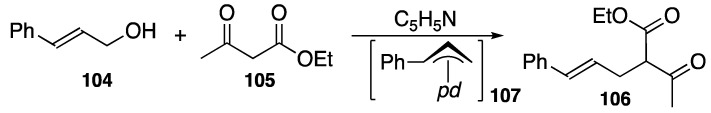
DPCB–palladium-catalyzed Tsuji–Trost-type alkylation reaction; *pd* = (DPCB)Pd^+^.

Direct alkylation can be regioselectively achieved using carbanions from active methylene compounds such as ethyl acetylacetate (**105**) as a nucleophile in order to formally substitute the OH group of allylic alcohol **104** with an alkylation product (**106**) in a high yield, as shown in Scheme 37 [112], where an intermediate **107** is supposed to be functional. It is not necessary to convert alcohol **104** to a more reactive derivative (such as an ester or halide) as an allylic starting material in advance of conventional Tsuji–Trost reactions.

**Scheme 38:**
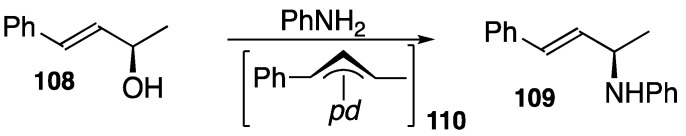
DPCB–palladium-catalyzed amination of chiral alcohol to amine; *pd* = (DPCB)Pd^+^.

Furthermore, the reaction from a chiral alcohol **108** of 98.5 %*ee* yields corresponding amine **109** of the same chirality in a 92% yield, which indicates that a double inversion process is involved and surrounds intermediate **110** during the reaction course to control the stereochemistry (Scheme 38) [112].

These results indicate that DPCB ligands have a high coordination ability due to their σ-donation/π-back donation interactions, strong π-acceptor property due to their low-lying LUMO (π*), and unique coordination structure with planarity. In addition, the catalysts are highly stable toward air and reusable after reactions in most cases. The above characteristics are superior to catalysts with ligands having sp^3^ phosphorus or sp^2^ nitrogen atoms for catalytic organic reactions.

**Scheme 39:**
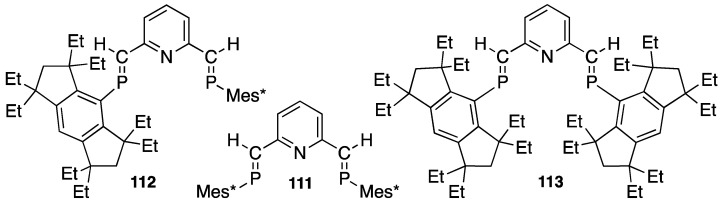
Some pincer ligands with sterically bulky P=C bonds (**111**–**113**).

Recently, Ozawa reported using pincer ligands such as **111**–**113** (Scheme 39) to activate CO bonds in carbon dioxide, taking advantage of the frustrated lone pair (FLP) within the ligand [113,114]. Further fundamental and applied investigation are expected to lead to progress in research of this type of low-coordinate phosphorus ligand for ideally efficient catalytic reactions.

## 5. Closing Remarks

Forty years have passed since a true “phosphobenzene” was prepared for the first time in 1981 via the steric protection methodology with an extremely bulky 2,4,6-tri-*t*-butylphenyl group (Mes*). It was believed, even theoretically, that molecules such as those with heavier main-group elements never existed as stable compounds, but various kinds of “unusual” phosphorus compounds have been isolated and characterized by utilizing the Mes* group. Interest in basic research on this class of compounds and synthetic applications for organic synthesis is high. This review article was based on a plenary lecture delivered by the author at the 23rd International Conference on Phosphorus Chemistry (ICPC-23) remotely held in Częstochowa, Poland, 5–9 July 2021 [115].

## Figures and Tables

**Table 1:** Some physical data of diphosphene **17**.

Item	Detailed Description	Ref.
MS	Found: 552.4012; Calcd: 552.4012	[12]
Mol. Wt. (C_6_H_6_)	552.1	[12]
UV (CH_2_Cl_2_)	*λ*_max_ (ε) 284 (15,660), 340 (7690), 460 (1360) nm	[12]
^1^H NMR (CCl_4_)	*δ* 7.30 (s, 4H, arom.), 1.45 (s, 36H, *o*-*t*-Bu), 1.35 (18H, *p*-*t*-Bu)	[12]
^31^P NMR (C_6_D_6_)	*δ*_P_ 492.4 ppm	[13]
Raman	*ν*(P=P) 610 cm^–1^	[14]
X-ray	P=P 2.034(2), P–C 1.862(2) Å; P–C–C 102.8(1), C–P–P–C 172.2(1)°	[12]

**Table 2:** ^31^P NMR data for some diphosphenes.

Diphosphene		*E*/Z	Chemical Shift *δ*_P_	Coupling Constant ^1^*J*_PP_/Hz	Ref.
Mes*–P=P–Mes*	(**17**)	*E*	492.4	–––	[12,13]
Mes*–P=P–2,4-*t*-Bu_2_-6-MeC_6_H_2_	(**18A**)	*E*	517.0; 480.1	583.5	[18]
Mes*–P=P–Mes	(**18B**)	*E*	540.4; 467.6	573.7	[18]
Mes*–P=P–Ph	(**18C**)	*E*	525.5; 455.5	548.7	[18]
Mes*–P=P–2,4,6-*t*-Pn_3_C_6_H_2_	(**18D**)	*E*	491.2; 489.9	582.9	[20]
Mes*–P=P–2,4,6-*i*-Pr_3_C_6_H_2_	(**18E**)	*E*	535; 470	572	[21]
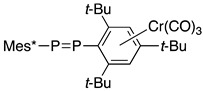	(**18F**)	*E*	503.2; 475.6	590.8	[22]
Mes*–P=P–C(SiMe_3_)_3_	(**18G**)	*E*	533.1; 530.0	619.7	[23]
Cp*–P=P–Cp*	(**19**)	*E*	504.0	–––	[24]
(Me_3_Si)_3_C–P=P–C(SiMe_3_)_3_	(**20**)	*E*	599.6	–––	[16,25]
Mes*–P=P–Mes*	(**21**)	*Z*	368	–––	[26,27]
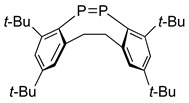	(**22**)	*Z*	394	–––	[28]

Mes = 2,4,6-Me_3_C_6_H_2_, Cp* = pentamethylcyclopentadienyl.

**Table 3:** ^31^P NMR for some phosphaalkenes, phosphaalkynes, and related compounds.

Compound		*δ*_P_/ppm	Ref.
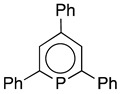	(**31**)	178.2	[43]
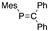	(**32**)	233.0	[44]
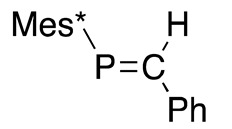	(**33**)	259.3	[45]
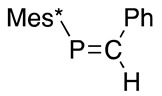	(**34**)	241.6	[45]
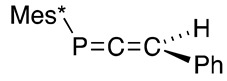	(**36**)	75.2	[47]
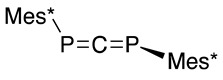	(**37**)	142.4	[48]
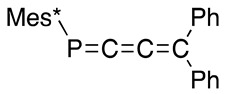	(**38**)	156.7	[49,50]
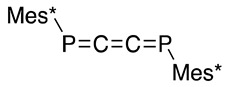	(**39**)	180.6	[51,52]
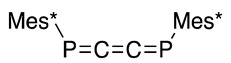	(**40**)	170.0	[51,52]
Mes*–P=C=NPh	(**42**)	−106.2	[53]
Mes*–P=C=O	(**43**)	−207.4	[54]
*t*-Bu–C≡P	(**45**)	−69.2	[55]
Mes*–C≡P	(**46**)	34.4	[56]

Mes = 2,4,6-Me_3_C_6_H_2_; Mes* = 2,4,6-*t*-Bu_3_C_6_H_2_.
